# Mechanism of Electroacupuncture Against Cerebral Ischemia–Reperfusion Injury: Reducing Inflammatory Response and Cell Pyroptosis by Inhibiting NLRP3 and Caspase-1

**DOI:** 10.3389/fnmol.2022.822088

**Published:** 2022-05-06

**Authors:** Li Cai, Zeng-Yu Yao, Lu Yang, Xiu-Hong Xu, Meng Luo, Miao-Miao Dong, Guo-Ping Zhou

**Affiliations:** ^1^Department of Acupuncture and Massage Rehabilitation, Neuroscience Center, Integrated Hospital of Traditional Chinese Medicine, Southern Medical University, Guangzhou, China; ^2^School of Traditional Chinese Medicine, Southern Medical University, Guangzhou, China

**Keywords:** nerve regeneration, cerebral ischemia/reperfusion injury, NLRP3, caspase-1, cell pyroptosis, electroacupuncture

## Abstract

Cell pyroptosis is one of the main forms of neuronal injury after cerebral ischemia–reperfusion. It is accompanied by an inflammatory reaction and regulated by the caspase gene family. Electroacupuncture (EA) can reduce neuronal injury caused by cerebral ischemia–reperfusion, and we speculated that EA can prevent neuronal pyroptosis after cerebral ischemia–reperfusion by regulating the nucleotide-binding oligomerization domain-like receptor protein 3 (NLRP3)/caspase-1 pathway. The cerebral ischemia–reperfusion injury model of C57 and caspase-1 gene knockout (Cas-1 ko) mice was established by Longa's method. EA was conducted at acupoints Chize (LU5), Hegu (LI4), Sanyinjiao (SP6), and Zusanli (ST36) for 1.5 h after cerebral ischemia–reperfusion injury for 20 min, and observation was carried out after 24 h. Neurological deficit scores evaluated the neurological function, cerebral infarction volume was observed by triphenyl tetrazolium chloride (TTC) staining, hematoxylin and eosin (H&E) staining, TUNEL and caspase-1 double-labeled fluorescence staining, and NLRP3 and caspase-1 double-labeled immunofluorescence staining that were used to observe the morphology of neurons in hippocampus, and the protein expression of NLRP3, pro-caspase-1, cleaved caspase-1 p20, pro-interleukin-1β (IL-1β), cleaved IL-1β, and GSDMD was detected by Western blot assay. Results showed that EA could reduce the score of neurological deficit, reduce the volume of cerebral infarction and improve the degree of nerve cell injury, and inhibit NLRP3, pro-caspase-1, cleaved caspase-1 p20, pro-IL-1β, cleaved IL-1β, and GSDMD protein expression. In summary, EA plays a neuroprotective role by reducing the pyroptotic neurons that were caspase 1-mediated and inflammatory response after cerebral ischemia–reperfusion.

## Background

Ischemic stroke is one of the most severe diseases that affect human health and death in the world. It has the characteristics of high morbidity, high disability rate, high mortality, and high recurrence rate (Dabrowska-Bender et al., 2017; Zhou et al., 2019). After a certain period of cerebral ischemia, blood flow is recanalized. However, a large amount of blood oxygen supply can further cause more severe nerve damage and accelerate the death of nerve cells. This phenomenon is called cerebral ischemia/reperfusion injury (Lv et al., 2020). The pathophysiological mechanism of cerebral ischemia/reperfusion injury is a series of complex nerve injury cascade reactions, and the death of nerve cells runs through the whole injury process. Pyroptosis is a programmed cell death with inflammatory response, which has been found and confirmed in recent years. Its classical pathway depends on the activation of caspase-1, and endogenous and exogenous stimulation signals act on inflammatory bodies through different pathways to activate caspase-1. It mediates the swelling and rupture of cell permeability and causes the release of intracellular substances and pro-inflammatory factors, such as interleukin-1β (IL-1β) and IL-18, amplifies local and systemic inflammatory responses, and induces cell pyroptosis (Bergsbaken and Fink, 2009; Doitsh et al., 2014; Jorgensen, 2015).

The activation of inflammatory bodies is a classical pathway for the regulation of cell pyroptosis. Nucleotide-binding oligomerization domain-like receptor protein 3 (NLRP3) recruits caspase-1 through adaptor proteins, such as apoptosis-associated speck-like protein containing CARD (ASC), and induces its precursor protease (pro-caspase-1) to produce autohydration reaction. Activated caspase-1 can effectively trigger downstream components to cause cell pyroptosis (Jin, 2010; Wellington et al., 2014; Rathinam, 2016). NLRP3, as the initiating factor of aseptic inflammatory response of the central nervous system after cerebral ischemia, induces cell pyroptosis, resulting in nerve cell injury, and plays a key role in the inflammatory cascade effect after cerebral ischemia/reperfusion (Fann et al., 2013; Walsh and Muruve, 2014).

Inducing inflammatory response is a major feature of cell pyroptosis different from apoptosis. It is found that the classical caspase-1 signaling pathway mediating pyroptosis is activated in stroke and aggravates brain injury, while inhibiting caspase-1 activation can reduce stroke injury and play a protective role (Kawaguchi et al., 2011; He et al., 2017). Electroacupuncture (EA) is beneficial to improve the symptoms of neurological deficit and restore limb motor function after cerebral ischemia/reperfusion injury. “Chize (LU5), Hegu (LI4)” and “Sanyinjiao (SP6), and Zusanli (ST36)” served as common acupoints in the clinical treatment of stroke and could improve the symptoms of neurological deficit (Zhang et al., 2012; Yang et al., 2015; Wang et al., 2016; Zhu et al., 2017). Our previous studies have found that EA can reduce apoptosis and improve neurological injury after cerebral I/R, but its mechanism needs further investigation (Wu et al., 2015, 2018; Lan et al., 2017).

In this study, the cerebral I/R injury models of C57 and caspase-1 gene knockout (Cas-1 ko) mice were established, and the neurological deficit scores, cerebral infarction area, pyroptosis index, and the expression of NLRP3/caspase-1/IL-1β were analyzed to explore the mechanism of EA on nerve cell protection after cerebral ischemia/reperfusion.

## Methods

### Animals

A total of 48 healthy C57BL/6 mice (half male and half female), weighing 20–25 g, and a total of 48 Casp-1 ko mice (half male and half female), weighing 20–25 g, were purchased from Cyagen Biosciences Co. Ltd. (Guangzhou, China; license number: SCXK (Su) 2018-0003). The experimental procedure followed the National Institutes of Health Guide for the Care and Use of Laboratory Animals (NIH Publications No. 8023, revised 1986). The mice were fed standard rodent chow and allowed free access to water. It was approved by the Animal Ethics and Welfare Committee of Southern Medical University of China (approval no. 2021-005). The temperature of the animal house was maintained at a room temperature between 20 and 22°C and a relative humidity of 65–70%. The C57 mice and the caspase knockout mice were randomized into Sham (Sham control) group, I/R (model) group, and EA (I/R + EA) group (*n* = 16 per group).

### Establishing Cerebral I/R Injury Mouse Model

The middle cerebral artery of the mice was blocked according to the modified Zea-Longa's method to induce cerebral I/R model (Longa et al., 1989). After anesthetizing the mice with 1% pentobarbital sodium (45 mg/kg) by intraperitoneal injection, the left common carotid artery, left internal carotid artery, external carotid artery, and vagus nerve were carefully exposed through the midline incision under the surgical microscope. A nylon wire of about 11 ± 0.5 mm was introduced into the internal carotid artery to occlude the middle cerebral artery until slight resistance was observed during insertion. After 30 min of occlusion, the blood supply of the ischemic area was restored by slowly drawing the thread, and the reperfusion was achieved. Mice in the Sham group underwent the same procedures, as described previously, but without arterial occlusion. The model evaluation criteria refer to Zea-Longa's 5-point evaluation method (0, no obvious defect; 1, failure to fully extend the contralateral forepaw; 2, circling to the opposite side; 3, falling to the opposite side; 4, loss of walking or consciousness) (Longa et al., 1989). The behavioral evaluation of mice for 2 h after operation, and the score of neurological deficit was 1–3, was included in the follow-up experiment; those that did not meet the above scoring criteria were unconscious and found subarachnoid hemorrhage during brain removal, considering that the model was failed and was not included in the experiment and supplemented randomly.

### Electroacupuncture Intervention

The mice in the EA group underwent EA stimulation for 1.5 h after I/R injury. Stainless steel acupuncture needles (diameter: 0.16 × 13 mm^2^; Suzhou Universal Acupuncture Medical Devices Co., Ltd., Suzhou, China) were inserted 2–3 mm into LU5, LI4, ST36, and SP6 acupoints of the paralyzed limb. The selection of acupoints and EA stimulation were made according to “experimental acupuncture” edited by Li (2007). The location of the selected acupoints was as follows: as for LU5, in the depression of the outer end of the transverse cubital crease, an acupuncture needle was inserted perpendicularly to a depth of 3 mm. As for LI4, located between first metacarpal bone and second metacarpal bone, an acupuncture needle was inserted perpendicularly to a depth of 1 mm; as for ST36, located at 5 mm below fibular head at outer lateral posterior knee and puncture, an acupuncture needle was inserted perpendicularly to a depth of 7 mm; as for SP6, located at the tip of the inner ankle of the posterior limb, a needle was inserted upward 10 mm and perpendicularly to a depth of 5 mm. The acupoints were stimulated for 20 min with a dilatational wave of frequency 5/10 Hz and intensity 2 mA using an EA instrument (model KWD-808I, Suzhou Universal Acupuncture Medical Devices Co., Ltd).

### Neurological Deficit Scores

At 24 h after the operation (before tissue sampling), the neurological deficit score of mice was evaluated by the Zea-Longa's 5-point evaluation method (Longa et al., 1989). The neurological deficit scores were defined as follows: score 0, no obvious defect; score 1, failure to fully extend the right forepaw; score 2, circling to the contralateral side; score 3, falling to the opposite side; and score 4, not spontaneously walking or loss of consciousness.

### Tissue Sampling

The samples were collected for 24 h after I/R injury. After excessive anesthesia (1% pentobarbital sodium, 45 mg/kg), rapid perfusion with 0.9% normal saline (NaCl) and 4% paraformaldehyde in phosphate-buffered saline (PBS) for 3–5 min eliminated the influence of blood factors. Then, the whole brain was dissected out of the cranial cavity immediately. In each group, 4 fresh brain tissues were taken for triphenyl tetrazolium chloride (TTC) staining, 8 tissues were placed in 4% paraformaldehyde solution for hematoxylin and eosin (H&E) and TUNEL staining, and 4 tissues were stored at −80°C in refrigerator for Western blot analysis.

### TTC Staining

Fresh brain tissue was stored at −20°C for 20 min and then sectioned every 2 mm in the coronal plane. The slices were placed in 2% TTC phosphate buffer and incubated in a 37°C water bath for 30 min in the dark. Slices were turned evenly every 10 min to make the slices even in contact with the TTC staining solution. After staining, the infarct was stained white, and the normal brain tissue was stained red. The AlphaEaseFC analyzer software (AlphaInnotech, San Leandro, CA, USA) was used to measure the infarct size, and the brain infarct volume percentage (BIVP) was calculated.

### Hematoxylin and Eosin Staining

Brain tissue sections were dewaxed with xylene and absolute ethanol two times each and were hydrated. After cleaning, they were stained using hematoxylin (Yuanmu Biotechnology Co., Ltd., Shanghai, China) for 10 min. 1% hydrochloric acid ethanol is used for differentiation, and 1/400 ammonia is used for bluing. After cleaning, they were stained with eosin (Huihong Reagent Co., Ltd., Hunan, China) for 5 min. Ethanol was gradually dehydrated. Xylene was transparent twice and sealed with neutral resin. The morphology of nerve cells in hippocampus under a microscope (Jiangnan Optical Instrument Group, Nanjing, China) was observed to judge the degree of nerve cell injury.

### Immunofluorescence Staining

Frozen sections were boiled in ethylenediaminetetraacetic acid (EDTA) antigen repair solution (pH 8.0) (Servicebio, Wuhan, CNH) for antigen retrieval and blocked with 3% bovine serum albumin for 30 min at room temperature. Then, the sections were incubated at 4°C overnight with primary antibodies against caspase-1 (1:50, Servicebio, Wuhan, CNH, GB11383) and NLRP3 (1:100, Servicebio, Wuhan, CNH, GB11300). Then, the sections were incubated with the corresponding secondary antibody at room temperature for 90 min. The sections were washed in PBS 3 times (5 min/time), and 4',6-diamidino-2-phenylindole (DAPI) (Servicebio, Wuhan, CNH, GB1012) was used for staining the dried section for 10 min. Apoptosis was detected by a fluorescein (FITC) TUNEL cell apoptosis detection kit (Servicebio, Wuhan, CNH, G1501-50T). Morphological changes in the cerebral tissues were observed under a microscope (Jiangnan Optical Instrument Group, Nanjing, China), and the images were collected for analysis (AlphaEaseFC). Two 90 × fields of view in the hippocampal CA1 area were randomly selected for photography. The number of positive cells was calculated according to the following formula: double-stained cells/total cells × 100%.

### Western Blot Assay

Hippocampus tissues were homogenized in Radio Immunoprecipitation Assay (RIPA) lysis buffer, centrifuged at 12,000 × *g* for 5 min, and then determined protein concentration in supernatants. Protein lysates were separated by 10% SDS-PAGE gels and then electrophoretically transferred onto polyvinylidene fluoride membranes (Millipore, Boston, USA). The membranes were blocked with 5% nonfat dry milk for 1 h and incubated with primary antibodies, namely, caspase-1 (1:1,000; Servicebio, Wuhan, CNH; GB11383), NLRP3 (1:1,000; Affinity, Wuhan, CNH; DF7438), and IL-1β (1:1,000; Affinity, Wuhan, CNH; AF5103) overnight at 4°C. The membranes were incubated with a corresponding secondary antibody (1:3,000; goat anti-rabbit/mouse IgG; Servicebio, Wuhan, CNH; GB23303) in tris-buffered saline and tween 20 (TBST) for 30 min. The blots were developed using enhanced chemiluminescence, and the intensity of the bands was measured using the AlphaEaseFC analyzer software (AlphaInnotech, San Leandro, CA, USA). The optical density value ratio of the target band to the internal reference served as the relative expression of the target protein.

### Statistical Analysis

The SPSS 20.0 software (IBM, Armonk, NY, USA) was used for statistical analysis. Quantitative data are expressed as the mean ± SD. For data conforming to normal distribution and homogeneity of variance, two-way analysis of variance (ANOVA) is used; during the pairwise comparison between groups, the least significant difference (LSD) is calculated using the *post-hoc* test. If the data do not meet the normal distribution, the Mann–Whitney nonparametric test is used for pairwise comparison, and the Kruskal–Wallis test is used for multiple comparisons. A *p*-value less than 0.05 was considered statistically significant.

## Results

### Effect of Electroacupuncture on the Neurological Function After I/R Injury

**Figure 1:**
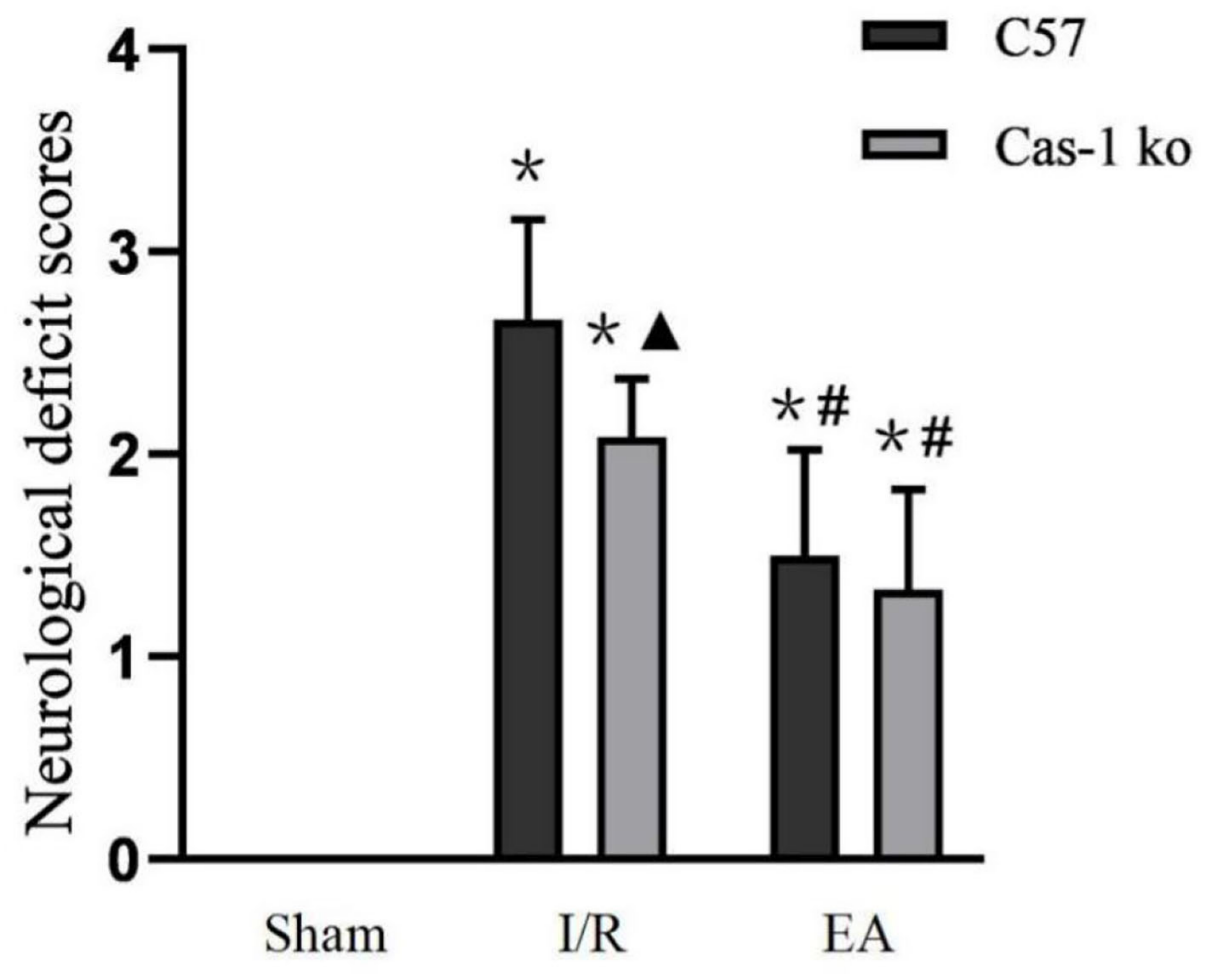
Neurological deficit scores. Comparison within the same genotype: *p < 0.05 vs. Sham group; ^#^*p* < 0.05 vs. I/R group. Comparison within the I/R group: ▴*p* < 0.05 vs. C57 mice. Data are representative of 12 independent experiments (mean ± SD are representative of values from 12 independent experiments).

The neurological deficits of mice in each group were evaluated. There was no nerve injury in the Sham group, but there was an obvious nerve function defect in the I/R and EA groups (*F* = 100.942, *p* = 0.026); in the I/R group, the neurological deficit score of Cas-1 ko mice was lower than that of C57 mice, but there was no significant difference between them in the EA group (*p* < 0.05). The neurological deficit score of the same genotype mice in the EA group was significantly lower than that in the I/R group (*p* < 0.05) (Figure 1).

### Effect of Electroacupuncture on Cerebral Infarction Volume Ratio After I/R Injury

**Figure 2:**
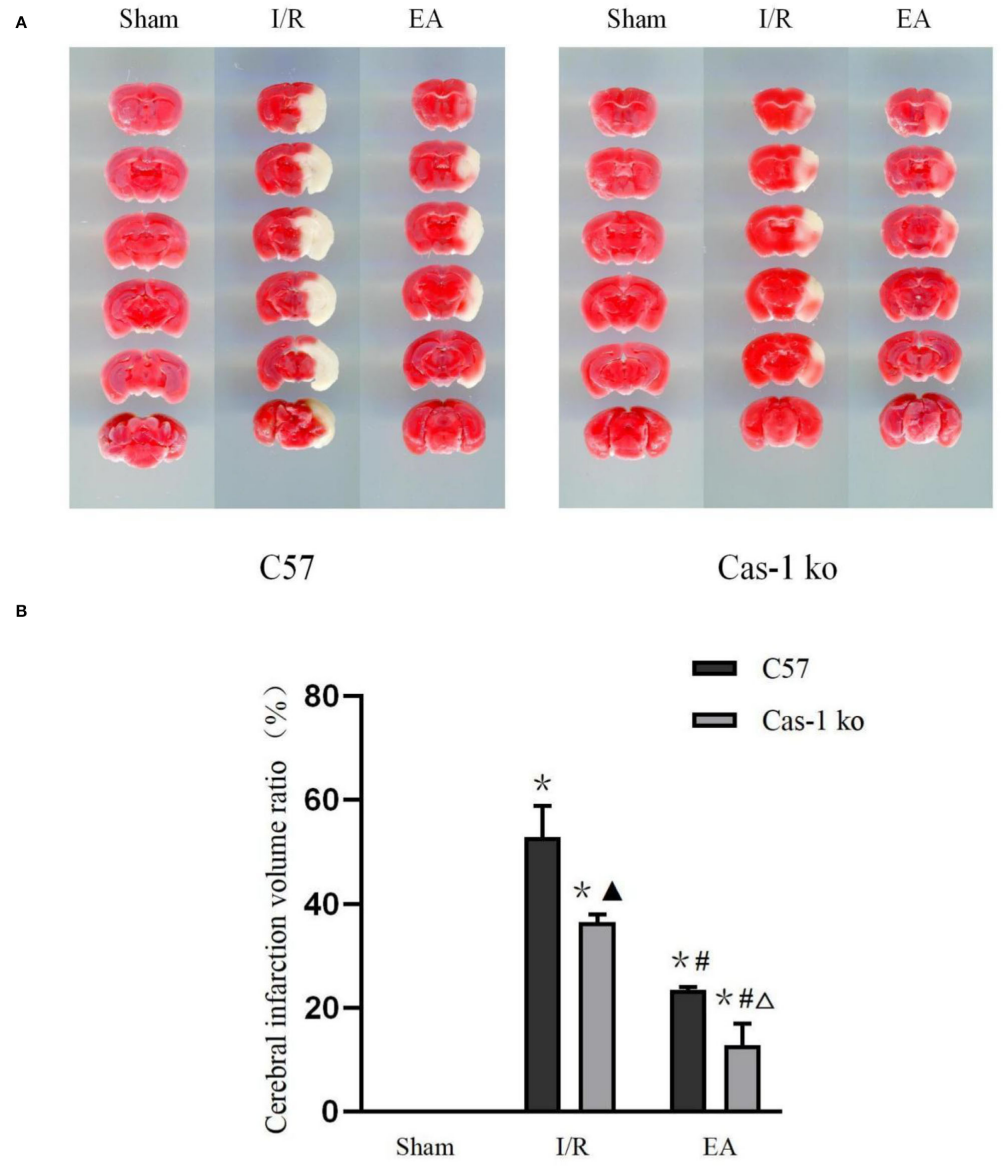
Brain tissue with TTC staining. **(A,B)** TTC staining results of different treatment groups and quantitative analysis of volume ratio. Comparison within the same genotype: *p < 0.05 vs. Sham group; ^#^*p* < 0.05 vs. I/R group. Comparison within the I/R group: ▴*p* < 0.05 vs. C57 mice. Comparison within the EA group: Δ*p* < 0.05 vs. C57 mice. Data are representative of four independent experiments [**(A,B)** mean ± SD are representative of values from four independent experiments in **(B)**].

The TTC staining showed that the brain tissue sections in the Sham group were bright red, and there was no pale infarct; pale infarcts of different sizes were observed in brain tissue sections of the I/R and EA groups (*F* = 194.386, *p* < 0.001). The volume of cerebral infarction in the I/R and EA groups was significantly higher than that in the Sham group, and that in the EA group was lower than that in the I/R group (*p* < 0.05). The cerebral infarction volume of Cas-1 ko mice in the I/R group was lower than that of C57 mice (*p* < 0.05), but there was no significant difference in cerebral infarction volume of different genotypes in the EA group (*P* > 0.05) (Figure 2).

### Morphological Observation of Electroacupuncture on Neuronal Injury in Hippocampus After I/R Injury

**Figure 3:**
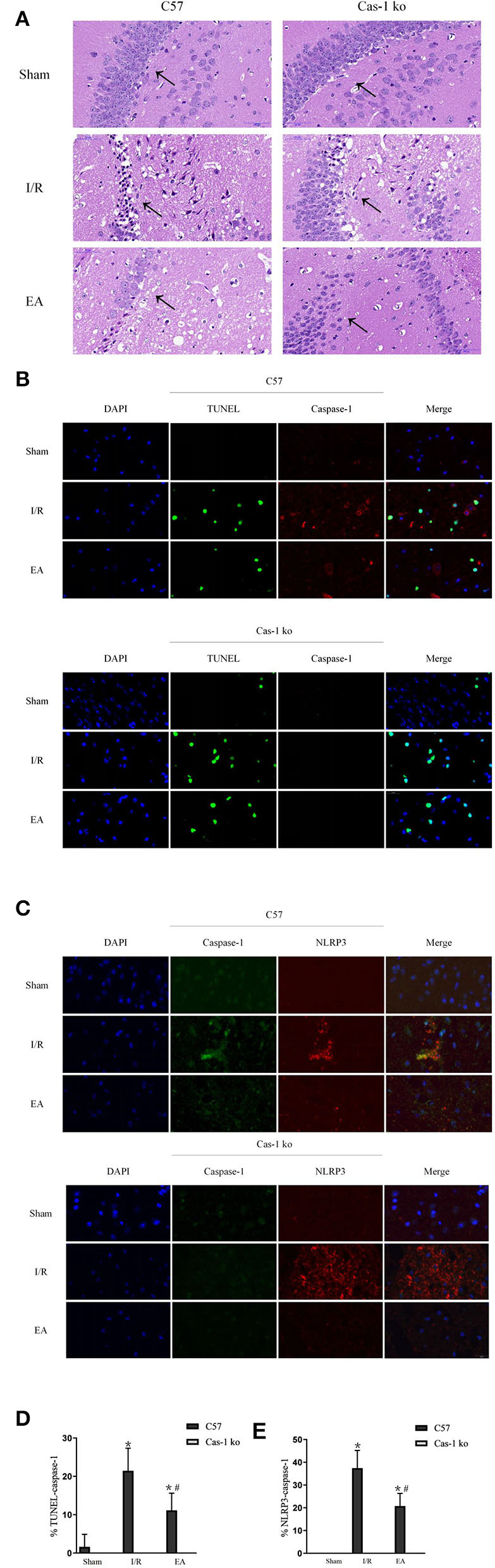
Morphological observation of neuronal injury in hippocampus after I/R injury. **(A)** Pathological HandE staining of neurons in hippocampus. **(B,D)** TUNEL (green)/caspase-1 (red) double-labeled staining of neurons in hippocampus and quantitative analysis of double-labeled cells. **(C,E)** Double immunofluorescent labeling with caspase-1 (green)/NLRP3 (red) and quantitative analysis of colocalization percentage. Comparison within the same genotype: *p < 0.05 vs. Sham group; ^#^*p* < 0.05 vs. I/R group. Comparison within the I/R group: ▴*p* < 0.05 vs. C57 mice. Comparison within the EA group: Δ*p* < 0.05 vs. C57 mice. Data are representative of four independent experiments [**(A–E)** mean ± SD are representative of values from four independent experiments in **(D,E)**].

The EA intervention has a cytoprotective effect on neurons in hippocampus after I/R injury. H&E staining showed that after I/R injury, the number of neurons decreased, the arrangement was loose, and vacuole-like changes and irregular nuclear morphology could be observed in the hippocampus. After EA intervention, the pathological changes of neurons in hippocampus were reduced, and less degeneration or necrosis of neurons was observed (Figure 3A). TUNEL staining could label the broken DNA fragments in apoptotic or pyroptotic cells (Figures 3B,D). In the I/R group, a large number of TUNEL and caspase-1 double-staining neurons were observed in C57 mice. After EA intervention, the positive rates in C57 mice decreased significantly (*p* < 0.05, Figures 3B,D). In addition, double-labeled immunofluorescence showed that the expression of caspase-1 and NLRP3 co-localization in neurons in the hippocampus of C57 mice was obvious and evident (*p* < 0.05, Figures 3C,E). EA intervention could effectively reduce the number of co-expressed neurons in C57 mice (*p* < 0.05, Figures 3C,E). There were no neurons co-expressing caspase-1 and NLRP3 in Cas-1 ko mice of different groups.

### Effect of Electroacupuncture on the Expression of Pyroptosis-Related Proteins in Mice After I/R Injury

**Figure 4:**
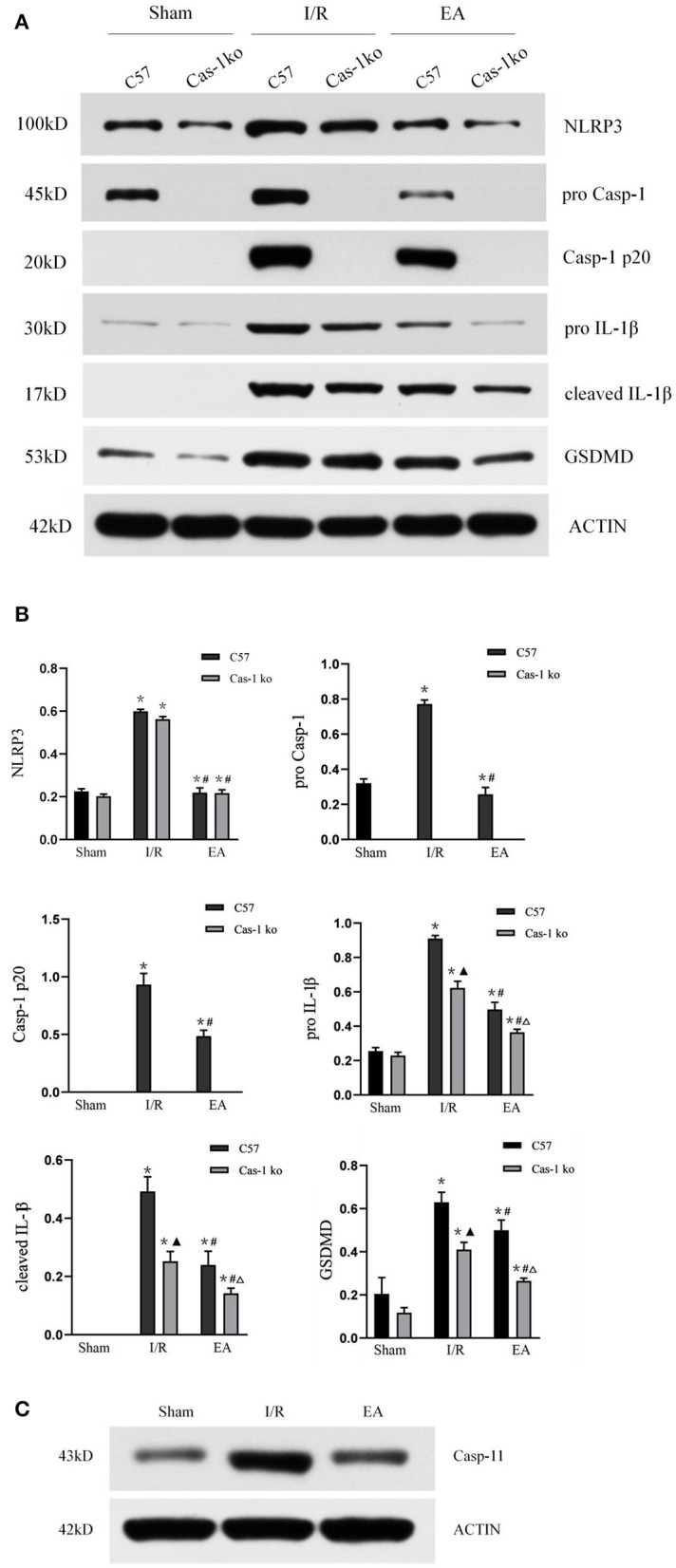
Protein expression of pyroptosis-related proteins. **(A,B)** Western blotting analysis of NLRP3, pro-Casp-1, Casp-1 p20, IL-1β, cleaved IL-1β, and GSDMD in different treatment groups and quantitative analysis of bands. **(C)** Western blotting analysis of casp-11 in Cas-1 ko mice. Comparison within the same genotype: *p < 0.05 vs. Sham group; ^#^*p* < 0.05 vs. I/R group. Comparison within the I/R group: ▴*p* < 0.05 vs. C57 mice. Comparison within the EA group: Δ*p* < 0.05 vs. C57 mice. Data are representative of two **(C)** or four independent experiments [**(A,B)** mean ± SD are representative of values from four independent experiments in **(B)**].

To explore the effect of EA on neuronal pyroptosis after I/R injury, brain protein was extracted, and the expression of NLRP3, pro-Casp-1, Casp-1 p20, IL-1β, cleaved IL-1β, and GSDMD was evaluated by Western blot. After I/R injury, the expression of NLRP3, pro-Casp-1, Casp-1 p20, IL-1β, cleaved IL-1β, and GSDMD in C57 mice increased significantly, while EA intervention inhibited the expression of those proteins (*p* < 0.05, Figures 4A,B). The expression of pro-Casp-1 and Casp-1 p20 of Cas-1 ko mice was absent in different treatment groups, but the trend of other proteins' expression in different treatment groups was similar to those in C57 mice. Furthermore, in the expression of casp-11 protein in Cas-1 ko mice, the results showed that Cas-1 ko mice could express casp-11 (Figure 4C).

## Discussion

A large number of clinical studies have confirmed that EA stimulation of acupoints can promote the recovery of stroke symptoms and paralyzed limb function, and can significantly improve the cognitive level and motor function of patients with ischemic stroke (Jittiwat, 2019; Nierhaus et al., 2019). Animal experiments showed that after cerebral ischemia, nerve function injury and cell death reached the peak on the first day, and then gradually decreased and stabilized (Kim et al., 2013a). EA intervention in the early stage after cerebral ischemia can increase cerebral blood flow, reduce the degree of cerebral ischemia/reperfusion injury, and expand the treatment time window (Ren et al., 2010; Kim et al., 2013b). This suggests that the best time for acupuncture treatment is within 3 h after ischemia. Therefore, we choose EA for 1.5 h after cerebral ischemia injury and 1 day after cerebral ischemia as the observation time. The neurological deficit score can effectively evaluate the behavioral changes after brain I/R injury, and it is also an important index to judge the recovery of tissue neurological function. Previous studies have shown that EA can improve limb motor function and has a certain neuroprotective effect on cerebral ischemia/reperfusion injury (Xue et al., 2014; Wu et al., 2017). The results of this study show that EA can significantly improve the neurological deficit symptoms after brain I/R injury. In the I/R group, caspase-1 gene mice obtain a lower neurological deficit score than C57 mice, which may be achieved by inhibiting cell death. After EA intervention, there was no difference between C57 mice and caspase-1 gene mice, suggesting that EA may play a neuroprotective role by inhibiting caspase-1. TTC staining of brain sections showed similar results. After brain I/R injury, C57 mice had larger cerebral infarction than Cas-1 ko mice. After EA intervention, the cerebral infarction volume of the two mice was significantly lower than that of the I/R group, and there was no significant difference between the two groups. Relevant studies have shown that inhibiting the expression of caspase-1 can reduce the neuronal injury in the brain after cerebral ischemia/reperfusion and reduce the cerebral infarct area after cerebral ischemia/reperfusion, such as knocking out the caspase-1 gene in mice or using the caspase-1 inhibitor Ac-YVAD = CMK (Liu et al., 2018; Liang et al., 2021). EA may play a similar role.

Under the light microscope, we detected the morphology of nerve cells in hippocampal CA1 area of mice brain by H&E staining, TUNEL+caspase-1 double-labeled staining, and NLRP3+caspase-1 immunofluorescence double staining. Pyroptosis is a form of cell death characterized by both apoptosis and necrosis in morphology and pathophysiology. Its most obvious feature is the rapid formation of plasma membrane pores, cell swelling and osmotic necrosis, and the release of a large number of cell contents and pro-inflammatory mediators to form excessive inflammatory response (Fink and Cookson, 2005). In the I/R group, H&E staining showed the typical characteristics of cell death, loose brain tissue structure, interstitial edema, disordered and swollen nerve cells, pyknosis, and fragmentation of nuclei, and the number of nerve cells decreased significantly. However, in the EA group, the occurrence of this phenomenon was reduced, and only a small amount of nerve cell degeneration and necrosis were observed in the peripheral area of ischemic focus. In TUNEL fluorescence staining, diaminobenzidine (DAB) was used as a marker to detect the active site of peroxidase in cells. Green or brownish-yellow granular precipitates could be formed in apoptotic and focal nuclei, while there was no broken DNA fragment in normal cells and would not react with the marker, so the normal nucleus was blue (Mirzayans and Murray, 2020). In addition, we added caspase-1 staining on the basis of TUNEL in order to identify the pyroptotic neurons which were caspase 1-mediated. In the I/R group, the positive rate of TUNEL and caspase-1 double-stained neurons in C57 mice significantly increased, which suggested that pyroptosis was involved in cerebral ischemia/reperfusion injury. After EA intervention, the positive rate in C57 mice significantly decreased, indicating that EA could reduce the pyroptosis of neurons in hippocampal CA1 area after brain I/R injury, which may be achieved by inhibiting the expression of caspase-1. Previous studies have also shown that acupuncture can inhibit neuronal apoptosis in hippocampus of rats with cerebral ischemia and can promote the recovery of neural cells after cerebral ischemia/reperfusion injury (Wang et al., 2008). Furthermore, double-labeled immunofluorescence showed that NLRP3 and caspase-1 were significantly co-expressed in neurons of C57 mice in the I/R group, which was similar to other studies (Sun et al., 2019; Liu et al., 2021). The number of co-expressed neurons in C57 mice in the EA group was reduced, which suggests that EA intervention may inhibit pyroptosis.

During cerebral ischemia/reperfusion injury, pyroptosis-related inflammatory bodies, such as nlrp1 and NLRP3, are highly expressed in glial cells and neurons. These activated inflammatory bodies activate caspase-1 and induce pyroptosis, which aggravates cerebral ischemia/reperfusion injury (Chavarría-Smith and Vance, 2015). The results of our Western blot verified this conclusion. In the I/R group, the NLRP3 level of C57 and Cas-1 ko mice was highly expressed, which was significantly different from that of the Sham group. Knockout of caspase-1 gene had no effect on the expression of NLRP3. Cas-1 ko mice expressed NLRP3 at the same level as C57 mice in the I/R group. Studies have shown that activated caspase-1 cleaves gsdmd protein and activates IL-18 and IL-1β precursors at the same time. The cleaved gsdmd protein binds to the inner lobules of the plasma membrane and oligomerizes, resulting in the formation of 10–20 nm pores in the cell membrane, and the release of mature IL-18, IL-1β, and other cell contents outside the cell, thus activating a strong inflammatory cascade (He et al., 2015; Wang et al., 2018). In this study, the expression of pro-caspase-1, cleaved caspase-1 p20, pro-IL-1β, cleaved IL-1β, and GSDMD in C57 mice after cerebral ischemia/reperfusion was significantly higher than those in Cas-1 ko mice in the same treatment group, suggesting that blocking the activation of caspase-1 can effectively inhibit the inflammatory response after cerebral ischemia/reperfusion. After EA intervention, the expression of NLRP3, pro-caspase-1, cleaved caspase-1 p20, pro-IL-1β, cleaved IL-1β, and GSDMD in C57 mice decreased significantly, and the expressions of NLRP3 in Cas-1 ko mice decreased significantly, indicating that EA could inhibit the expression of NLRP3, pro-caspase-1, cleaved caspase-1 p20, pro-IL-1β, cleaved IL-1β, and GSDMD in hippocampus after cerebral ischemia/reperfusion. IL-1β plays an important role in promoting inflammation. Under the pathological conditions of ischemia and hypoxia, it becomes active IL-1β after being cut by caspase-1, induces the activation and adhesion of inflammatory cells, leads to microvascular obstruction, and intensifies the inflammatory response (Davis et al., 2011; Man and Kanneganti, 2015). Therefore, the above results suggest that the neuroprotective effect of EA on cerebral ischemia/reperfusion injury reduces the pyroptosis of nerve cells by inhibiting the expression of NLRP3 and caspase-1, thereby reducing the release of inflammatory factors, such as IL-1β, so as to reduce the inflammatory response.

Studies by Man et al. (2017) described that having a deletion of caspase-1 leads to deletion of caspase-11. Cas-1 ko mice used in this study are C57BL/6N background. Theoretically, caspase-11 is normally expressed and the results of Western blot also confirmed the same. In addition, we also noticed that in the results of cerebral infarction volume measurement and TUNEL staining, there were differences between C57 mice and Cas-1 ko mice in the EA group, and the only factor causing these differences was caspase-1 knockout. Combined with the protein electrophoresis data of C57 mice, EA can inhibit the expression of caspase-1, but it cannot completely block the gene as Cas-1 ko mice. Interestingly, there was no significant difference between the neurological function score and the staining results of different genotypes in the EA group. We speculated that EA could not only inhibit the occurrence of pyroptosis, but also play a neuroprotective role in other ways. Our previous studies have confirmed that EA can upregulate the p-ERK protective pathway and inhibit p38MAPK apoptosis pathway after cerebral ischemia–reperfusion in mice (Wu et al., 2015, 2018; Lan et al., 2017). These mechanisms can be used as an evidence of our above hypothesis, that is, EA can resist cerebral ischemia–reperfusion injury in a variety of ways.

In conclusion, EA at points “LU5,” “LI4,” “ST36,” and “SP6” can significantly improve the symptoms of nerve injury and morphological changes of brain cells in mice with cerebral ischemia/reperfusion injury, reduce the infarct area, and protect brain tissue from ischemic injury. This treatment reduced the expression of NLRP3, caspase-1, and IL-1β, and the occurrence of cell pyroptosis and inflammatory response, suggesting that the protective effect of EA on cerebral ischemia/reperfusion injury may be played by inhibiting the pyroptosis pathway dependent on caspase-1.

## Data Availability Statement

The original contributions presented in the study are included in the article/supplementary materials, further inquiries can be directed to the corresponding author/s.

## Ethics Statement

The animal study was reviewed and approved by Animal Ethics and Welfare Committee of Southern Medical University of China.

## Author Contributions

G-PZ obtained funding and participated in study concept and design, and paper authorization. X-HX and ML purchased animals and material instruments. Z-YY analyzed data. LC wrote the paper. M-MD operated the acupuncture. LY ensured the integrity of the data. All authors approved the final version of the article.

## Funding

This study was supported by the National Natural Science Foundation of China, (No. 81674048). The funding body played no role in the study design, in the collection, analysis, and interpretation of data, in the writing of the report, or in the decision to submit the article for publication.

## Conflict of Interest

The authors declare that the research was conducted in the absence of any commercial or financial relationships that could be construed as a potential conflict of interest.

## Publisher's Note

All claims expressed in this article are solely those of the authors and do not necessarily represent those of their affiliated organizations, or those of the publisher, the editors and the reviewers. Any product that may be evaluated in this article, or claim that may be made by its manufacturer, is not guaranteed or endorsed by the publisher.
